# Effects of hypoxia on the biological characteristics of urine-derived stem cells

**DOI:** 10.3389/fcell.2026.1851623

**Published:** 2026-07-27

**Authors:** Yuzhen Xiao, Ziliang Guo, Wenze Shao, Huiqi Xie, Yizhou Huang

**Affiliations:** 1 Department of Orthopedic Surgery and Orthopedic Research Institute, Stem Cell and Tissue Engineering Research Center, State Key Laboratory of Biotherapy, West China Hospital, Sichuan University, Chengdu, China; 2 West China School of Basic Medical Sciences and Forensic Medicine, Sichuan University, Chengdu, China; 3 Division of Thyroid and Parathyroid Surgery, West China Hospital, Sichuan University, Chengdu, China; 4 Sports Medicine Center, West China Hospital, Sichuan University, Chengdu, China

**Keywords:** biological characteristics, hypoxia, regenerative medicine, tissue engineering, urine-derived stem cells

## Abstract

**Introduction:**

Urine-derived stem cells (USCs) are a promising stem-cell source because they can be obtained non-invasively and are readily available. However, their biological characteristics under hypoxic conditions remain insufficiently characterized. This study evaluated the effects of hypoxia on USCs *in vitro*.

**Methods:**

USCs isolated from six healthy male donors were cultured under normoxic (21% O2) or hypoxic (3% O2) conditions. Colony formation, viability, proliferation, cell cycle, apoptosis, migration, surface markers, stemness-related gene expression, multilineage differentiation, cytokine secretion, and transcriptomic changes were assessed.

**Results:**

Hypoxia enhanced colony formation, proliferation, migration, stemness-related gene expression, and osteogenic, adipogenic, and chondrogenic differentiation, while maintaining cell viability and not significantly affecting apoptosis or surface marker expression. VEGF secretion increased, whereas other assessed cytokines showed no significant differences. RNA sequencing identified 1,267 differentially expressed genes, including 748 upregulated and 519 downregulated genes. Hypoxia upregulated oxygen-sensing, metabolic, and HIF-1 pathways and downregulated Hippo signaling.

**Discussion:**

Hypoxia at 3% O2 enhanced multiple biological characteristics of USCs without compromising viability. These findings provide a foundation for further investigation of hypoxia-conditioned USCs in regenerative medicine.

## 1 Introduction

Stem cells are integral to the field of regenerative medicine, offering promising therapeutic potential for a wide range of diseases and conditions. Among the various types of mesenchymal stem cells (MSCs), those derived from bone marrow, adipose tissue, and umbilical cord blood are most frequently utilized in preclinical studies and clinical trials (Levy et al., 2020). However, the broader clinical use of these cells, particularly for autologous transplantation, is often constrained by challenges such as limited availability, invasive harvesting procedures, and complex sourcing processes. The identification of urine-derived stem cells (USCs) presents a promising alternative to address these limitations (Huang et al., 2022). USCs exhibit strong self-renewal capacities and favorable multi-differentiation potential (Zhang et al., 2008). Studies have demonstrated that USCs can differentiate into various cell types, including osteoblasts, adipocytes, chondrocytes, urothelial cells, smooth muscle cells, and cardiomyocytes (Bharadwaj et al., 2013; Guan et al., 2014). Furthermore, the non-invasive, straightforward and cost-effective isolation of USCs enhances their suitability for future clinical applications, establishing them as a versatile and practical resource in regenerative medicine (Fan et al., 2024; Atia et al., 2024).

Hypoxia is a well-documented microenvironmental factor that significantly influences tissue repair and regeneration. Unlike the atmospheric oxygen tension typically encountered *in vitro* (approximately 21% O_2_), the oxygen tension *in vivo* is markedly lower, often decreasing to 3% or even less, especially in injured tissues (Csete, 2005; Ceradini et al., 2004). Investigating the impact of hypoxia on stem cells is crucial for advancing their clinical applications in tissue regeneration. Previous research has shown that hypoxia changes stem cell proliferation, mobilization, migration, and angiogenic capacity, while also modulating their differentiation potential (Das et al., 2010). Specifically, in MSCs, hypoxia has been shown to promote proliferation (Wang et al., 2022), migration (Yang et al., 2019), and angiogenesis (Zhu et al., 2014). However, the effect of hypoxia on MSC differentiation, including osteogenic (Ciapetti et al., 2016; Yang et al., 2019), chondrogenic (Duval et al., 2012; Leijten et al., 2014), and adipogenic (Valorani et al., 2012) differentiation, remains contentious, with outcomes differing across various studies. Although some inconsistencies persist due to variable hypoxic conditions and MSC sources, the overall positive effects of hypoxic culture conditions underscore the potential utility of hypoxic preconditioning for MSCs in tissue regeneration.

Our prior research indicated that hypoxic preconditioning of USCs not only upregulated the expression of genes associated with angiogenesis and wound re-epithelialization but also augmented the secretion of growth factors (Zhang et al., 2020). However, the impact of hypoxia on USCs remains a subject of debate. Several studies have reported that hypoxic preconditioning enhances USC self-renewal, migration, colony formation, mitochondrial function, and exosome-mediated angiogenesis (Hu et al., 2023; Hu et al., 2021). However, emerging evidence suggests that hypoxia may not uniformly enhance differentiation potential, with outcomes potentially influenced by oxygen concentration and exposure duration (Alwohoush et al., 2024). In this study, we evaluated the survival, proliferation, migration, differentiation, and paracrine action of USCs cultured under 3% oxygen tension. Furthermore, we performed differential gene expression analysis to elucidate the effects of hypoxia on the USC transcriptome. This study seeks to contribute to the growing body of knowledge about USC biology, and further establishes a foundation for its future applications in tissue repair.

## 2 Materials and methods

### 2.1 Cell culture

This study received approval from the Institutional Review Board and Ethics Committee of West China Hospital, Sichuan University. Fresh mid-stream urine samples were obtained from six healthy male donors aged between 7 and 30 years. USCs were isolated following previously established protocols (Zhang et al., 2020). Briefly, urine samples were centrifuged at 1,500 rpm for 10 min at room temperature, and the resulting cell pellets were washed twice with phosphate-buffered saline (PBS). Subsequently, the cells from the pellets were seeded into cell culture flasks and maintained in cell growth medium at 37 °C with 5% CO_2_. The cell growth medium consisted of 50% Keratinocyte Serum-Free Medium (Gibco, USA), 33.75% Dulbecco’s modified Eagle medium (DMEM, Gibco, USA), 11.25% Ham’s F-12 Nutrient Mixture (Gibco, USA), 5% fetal bovine serum (FBS, Gibco, USA), 5 ng/mL epidermal growth factor (EGF, Gibco, USA), 50 ng/mL bovine pituitary extract (Scienceu, USA), 0.4 μg/mL hydrocortisone (Sigma, USA), 5 μg/mL transferrin (Sigma, USA), 5 ng/mL bovine insulin (Sigma, USA), 0.18 mM adenine (Sigma, USA), 2 nM 3,3′,5-triiodo-L-thyronine (Sigma, USA), 100 units/mL penicillin (Gibco, USA), and 100 μg/mL streptomycin (Gibco, USA). After an initial cell seeding period of 5–7 days, the medium was replaced to remove non-adherent cells and subsequently refreshed twice per week. Upon reaching 80% confluence, the cells were passaged at a ratio of 1 : 3. Cells at passages 3 to 5 were utilized for experimental purposes. Each experiment was independently repeated 2–5 times using USC samples derived from different donors. For flow cytometric analysis, USC samples from four independent donors were analyzed separately, and the resulting data were then combined for statistical analysis.

### 2.2 Colony formation assay

To evaluate the colony formation capacity of USCs, a total of 2,000 cells were seeded into each 25 cm^2^ flask and incubated under either normoxic (21% O_2_) or hypoxic (3% O_2_) conditions for a duration of 7 days, with five replicates in each group. Colony formation was assessed through crystal violet staining and visualized using an optical microscope (Nikon ECLIPSE Ti2-U, Japan). In brief, the growth medium was removed, and the cells were fixed with 4% paraformaldehyde for 30 min at room temperature. Subsequently, the cells were stained with 0.5% crystal violet (Beyotime, China) for 30 min. After washing the cells two to three times with PBS, the colonies were photographed. The colony formation efficiency (CFE) was determined using the formula: CFE = (Number of Colonies/2000) × 100%.

### 2.3 Live/dead staining

USCs were seeded at a density of 2 × 10^4^ cells per well in a 24-well plate and cultured under normoxic conditions (21% O_2_) or hypoxic conditions (3% O_2_) for a duration of 3 days, with four replicates per group (n = 4). Following the incubation period, the cells were subjected to staining using the Calcein-AM/PI Double Stain Kit (YEASEN, China) in accordance with the manufacturer’s instructions. Briefly, cells were harvested, washed 2–3 times with 1× Assay Buffer, and subsequently incubated with the staining solution at a ratio of 2 : 1 (v/v) at 37 °C for 15 min. Fluorescence was examined using an inverted fluorescence microscope (IX73, Olympus, Japan).

### 2.4 DNA content assay

USCs were seeded at a density of 1 × 10^4^ cells/cm^2^ and cultured under normoxic (21% O_2_) or hypoxic (3% O_2_) conditions, with four replicates in each group. Genomic DNA was extracted on days 3, 5, and 7 utilizing the Universal Genomic DNA Kit (CW2298S, cwbio, China) in accordance with the manufacturer’s instructions. Briefly, the cells were collected and lysed in GTL buffer containing Proteinase K, followed by the addition of GL buffer and ethanol. The resulting lysate was applied to spin columns, washed sequentially with GW1 and GW2 buffers, and eluted with GE buffer to obtain purified genomic DNA. The DNA concentration was quantified using a NanoDrop spectrophotometer.

### 2.5 Cell cycle assay

USCs were seeded at a density of 1 × 10^4^ cells/cm^2^ and incubated under normoxic (21% O_2_) or hypoxic (3% O_2_) conditions for a duration of 3 and 5 days, with four replicates in each experimental group. Subsequently, the cells were harvested, washed twice with PBS, and fixed in 75% ice-cold ethanol for two hours. Cell cycle analysis was conducted using the Cell Cycle and Apoptosis Analysis Kit (Beyotime, China) according to the manufacturer’s instructions. Data acquisition was performed using a flow cytometer (Beckman CytoFLEX, USA).

### 2.6 Cell apoptosis assay

USCs were cultured at a density of 1 × 10^4^ cells/cm^2^ in culture flasks and subjected to either normoxic or hypoxic conditions for a duration of 3 days, with four replicates in each experimental group. Then, cells were harvested, washed twice with PBS, resuspended in Binding Buffer, and stained according to the manufacturer’s instructions using the Annexin V-FITC/PI Apoptosis Detection Kit (YEASEN, China). Apoptotic cell populations were quantified utilizing a flow cytometer (BD FACSAria III, USA).

### 2.7 Cell migration assay

USCs were cultured in a 6-well plate at a density of 1 × 10^4^ cells/cm^2^, with five replicates per experimental group. Upon reaching full confluency, the cells were subjected to overnight starvation in DMEM supplemented with 0.5% FBS. A scratch was introduced using a 1 mL pipette tip, and PBS was employed to remove cellular debris. Subsequently, fresh culture medium containing 0.5% FBS was added, and the plates were incubated under either normoxic (21% O_2_) or hypoxic (3% O_2_) conditions. Microscopic images were captured at 0, 12, 24, and 48 h using an optical microscope (Nikon ECLIPSE Ti2-U, Japan) and subsequently analyzed with ImageJ software (National Institutes of Health, USA, https://imagej.net/ij/). The rate of scratch healing was quantified using the formula: Scratch Healing Rate = [(Initial Scratch Area at 0 h - Scratch Area at Subsequent Time Point)/Initial Scratch Area at 0 h] × 100%.

### 2.8 Cell surface marker analysis

USCs were cultured in 75 cm^2^ flasks at a density of 1 × 10^4^ cells/cm^2^ and incubated under either normoxic (21% O_2_) or hypoxic (3% O_2_) conditions for a duration of 3 days. Following incubation, the cells were collected, washed once with PBS, and centrifuged at 400 *g* for 5 min. The resulting cell pellet was resuspended in PBS and adjusted to a concentration of 2 × 10^5^ cells/mL. For surface marker analysis, 100 µL of the cell suspension was mixed with fluorochrome-conjugated mouse anti-human antibodies (BioLegend). The antibodies employed included: PE-CD29 (Cat. No.: 303003), FITC-CD73 (Cat. No.: 344015), FITC-CD34 (Cat. No.: 343503), and PE-CD45 (Cat. No.: 304007). Following a 15-min incubation period with the antibodies, 1 mL of PBS was added, and the cells were centrifuged at 400 *g* for 5 min. The supernatant was discarded, and the cell pellet was resuspended in 300 µL of PBS. The suspension was then transferred to flow cytometry tubes for subsequent analysis. Data acquisition was conducted using a flow cytometer (BD FACSAria III, USA).

### 2.9 Reverse transcription quantitative polymerase chain reaction (RT-qPCR)

USCs were seeded at a density of 3 × 10^4^ cells/cm^2^ in 12-well plates, with three wells per group. The cells were maintained under either normoxic (21% O_2_) or hypoxic (3% O_2_) conditions for a duration of 3 days. After the culture period, total RNA was isolated employing the FastPure® Cell/Tissue Total RNA Isolation Kit V2 (Vazyme, China). A total of 1 μg of RNA was then reverse-transcribed into complementary DNA (cDNA) using the HiScript III All-in-one RT SuperMix (Vazyme, China). Quantitative polymerase chain reaction (qPCR) was conducted utilizing the Taq Pro Universal SYBR qPCR Master Mix (Vazyme, China). The primer sequences used for each target gene are detailed in Table 1. Beta-actin served as the housekeeping gene. Relative gene expression levels were determined using the 2^−ΔΔCt^ method.

**TABLE 1:** Primer sequences used for RT-qPCR

Gene	Forward primer (5′–3′)	Reverse primer (3′–5′)
*c-Myc*	GCCGGCTAGGGTGGAAGA	CCT​TGC​TCG​GGT​GTT​GTA​AGT​T
*Nanog*	AAT​ACC​TCA​GCC​TCC​AGC​AGA​TG	TGC​GTC​ACA​CCA​TTG​CTA​TTC​TTC
*Oct4*	GTA​TTC​AGC​CAA​ACG​ACC​ATC	CTG​GTT​CGC​TTT​CTC​TTT​CG
*Sox2*	GGG​AAA​TGG​GAG​GGG​TGC​AAA​AGA​GG	TTG​CGT​GAG​TGT​GGA​TGG​GAT​TGG​TG
*CCN2*	AAA​TGC​TGC​GAG​GAG​TGG​GT	TGT​CTT​CCA​GTC​GGT​AAG​CCG
*WTIP*	GTG​CGA​GAC​TAT​CAC​ACG​GT	AAC​GAC​GAC​ACA​GTA​GGT​GG
*AXIN2*	TCG​GGA​GCC​TAA​AGG​TCG​T	TCT​CAC​TGT​CGT​TGG​CGC​T
*NF2*	AAT​GCT​GAA​GAG​GAG​CTG​GT	CGT​AAG​AAG​CCA​GGA​GCA​CA
*HK2*	GAC​TTC​CGC​ACA​GAA​TTT​GAT​G	GAA​TGT​TAC​GGA​CAA​TCT​CAC​CC
*ENO2*	CAA​GGG​AGT​CAT​CAA​GGA​CAA​AT	AAA​CTC​TGA​GGC​AGC​AAC​ATC​C
*SLC2A1*	GCT​TCT​CCA​ACT​GGA​CCT​CAA​A	GAA​GAA​CAG​AAC​CAG​GAG​CAC​AG
*PFKP*	CAG​CTT​GCG​TCG​TGT​CAC​TG	TGA​TGG​CAA​GTC​GCT​TGT​AGG
*β-actin*	AGCCTCGCCTTTGCCGA	CTGGTGCCTGGGGCG
*RUNX2*	CCAACCCACGAATGCACTATC	TAGTGAGTGGTGGCGGACATAC
*OPN*	GGT​GAT​AGT​GTG​GTT​TAT​GG	TGATGTCCTCGTCTGTAG
*COL1A1*	AGG​TGC​TGA​TGG​CTC​TCC​T	GGA​CCA​CTT​TCA​CCC​TTG​T
*Osterix*	GCC​AGA​AGC​TGT​GAA​ACC​TC	GCT​GCA​AGC​TCT​CCA​TAA​CC
*SOX9*	AGC​GAA​CGC​ACA​TCA​AGA​C	CTG​TAG​GCG​ATC​TGT​TGG​GG
*COL2A1*	AGCAAGAGCAAGGAGAAG	GGGAGCCAGATTGTCATC
*ACAN*	TCGAGGACAGCGAGGCC	TCG​AGG​GTG​TAG​CGT​GTA​GAG​A
*COMP*	AGA​AGT​CCT​ATC​GTT​GGT​TCC	CAAGACCACGTTGCTGTC
*PPARG*	AGC​CTG​CGA​AAG​CCT​TTT​GGT​G	GGC​TTC​ACA​TTC​AGC​AAA​CCT​GG
*FABP4*	ACG​AGA​GGA​TGA​TAA​ACT​GGT​GG	GCG​AAC​TTC​AGT​CCA​GGT​CAA​C
*CEBPA*	AGG​AGG​ATG​AAG​CCA​AGC​AGC​T	AGT​GCG​CGA​TCT​GGA​ACT​GCA​G
*ADIPOQ*	CAG​GCC​GTG​ATG​GCA​GAG​ATG	GGT​TTC​ACC​GAT​GTC​TCC​CTT​AG

### 2.10 Multilineage differentiation

USCs were cultured in a 24-well plate at a density of 2 × 10^4^ cells per well, with five wells allocated to each group. Following cells adhension and attainment of approximately 90% confluence, the culture medium was replaced with the corresponding induction medium, and the plates were incubated under either normoxic (21% O_2_) or hypoxic (3% O_2_) conditions. For osteogenic differentiation, the human mesenchymal stem cell osteogenic induction differentiation kit (Cat. No.: HUXXC-90021, OriCell®, Sayer Biotech, China) was utilized. After a 28-day induction period, the cells underwent Alizarin Red staining. For adipogenic differentiation, the human mesenchymal stem cell adipogenic induction differentiation kit (Cat. No.: HUXXC-90031, OriCell®, Sayer Biotech, China) was employed, and following a 21-day induction period, the cells were stained with Oil Red O. For chondrogenic differentiation, the human mesenchymal stem cell chondrogenic induction differentiation kit (Cat. No.: HUXXC-90041, OriCell®, Sayer Biotech, China) was used, with Alcian Blue staining performed after a 28-day induction period. After staining, the samples were imaged using an inverted microscope (Nikon, Japan), and the areas of positive staining were quantified using ImageJ software (National Institutes of Health, USA, https://imagej.net/ij/) to determine the relative areas of calcium nodules, lipid droplets, and cartilage matrix.

To further validate lineage-specific differentiation at the molecular level, parallel samples were collected after 14 days of induction for RT-qPCR analysis. Osteogenic markers, including RUNX2, OPN, COL1A1, and Osterix; adipogenic markers, including PPARG, FABP4, CEBPA, and ADIPOQ; and chondrogenic markers, including SOX9, COL2A1, ACAN, and COMP, were examined. Total RNA extraction, reverse transcription, and RT-qPCR analysis were performed as described in Section 2.9. The primer sequences used for each target gene are detailed in Table 1. Beta-actin was used as the housekeeping gene, and relative gene expression levels were calculated using the 2^−ΔΔCt^ method.

### 2.11 Detection of cytokines

USCs were initially seeded at a density of 1 × 10^4^ cells/cm^2^ in 6-well plates and maintained in growth medium until they achieved approximately 90% confluency. Subsequently, the cells underwent three washes with PBS, followed by the addition of fresh culture medium devoid of FBS to each well. The plates were then incubated under either normoxic conditions (21% O_2_) or hypoxic conditions (3% O_2_) for a duration of 48 h. After incubation, the cell culture supernatants from each well were collected individually, and the DNA content was quantified for normalization purposes. The secretion profile of cytokines was assessed by Wayen Biotechnology Co. Ltd (Shanghai, China) utilizing the cytokine antibody array (Bio-Plex Pro Human Cytokine Group I Panel (27-plex)), in accordance with the manufacturer’s protocol. Fluorescence detection was performed using a Luminex system. The amount of cytokine was normalized to the DNA content, and the normalized datasets were subjected to statistical analysis.

### 2.12 Transcriptome sequencing and analysis

USCs were seeded at a density of 2 × 10^4^ cells/cm^2^ in culture flasks, with three replicates per group. The cells were maintained under either normoxic (21% O_2_) or hypoxic (3% O_2_) conditions until they reached approximately 90% confluency. For RNA sequencing, the samples were lysed in Trizol reagent (Invitrogen, USA) and subsequently stored at −80 °C until analysis. Transcriptome sequencing was performed by Wayen Biotechnologies Co. Ltd (Shanghai, China). Differentially expressed genes (DEGs) were identified using the DESeq2 software, applying a threshold of fold-change >2 and p < 0.05. The identified DEGs were then subjected to clustering analysis, as well as Gene Ontology (GO) and Kyoto Encyclopedia of Genes and Genomes (KEGG) pathway enrichment analyses.

### 2.13 Statistical analysis

Quantitative data were presented as mean ± standard deviation. Statistical analyses were conducted utilizing GraphPad Prism 10 software (GraphPad, San Diego, CA). A Student’s t-test was employed to assess differences between the normoxic and hypoxic groups, with a p-value of less than 0.05 considered indicative of statistical significance.

## 3 Results

### 3.1 Hypoxia enhances the colony formation ability of USCs

**FIGURE 1:**
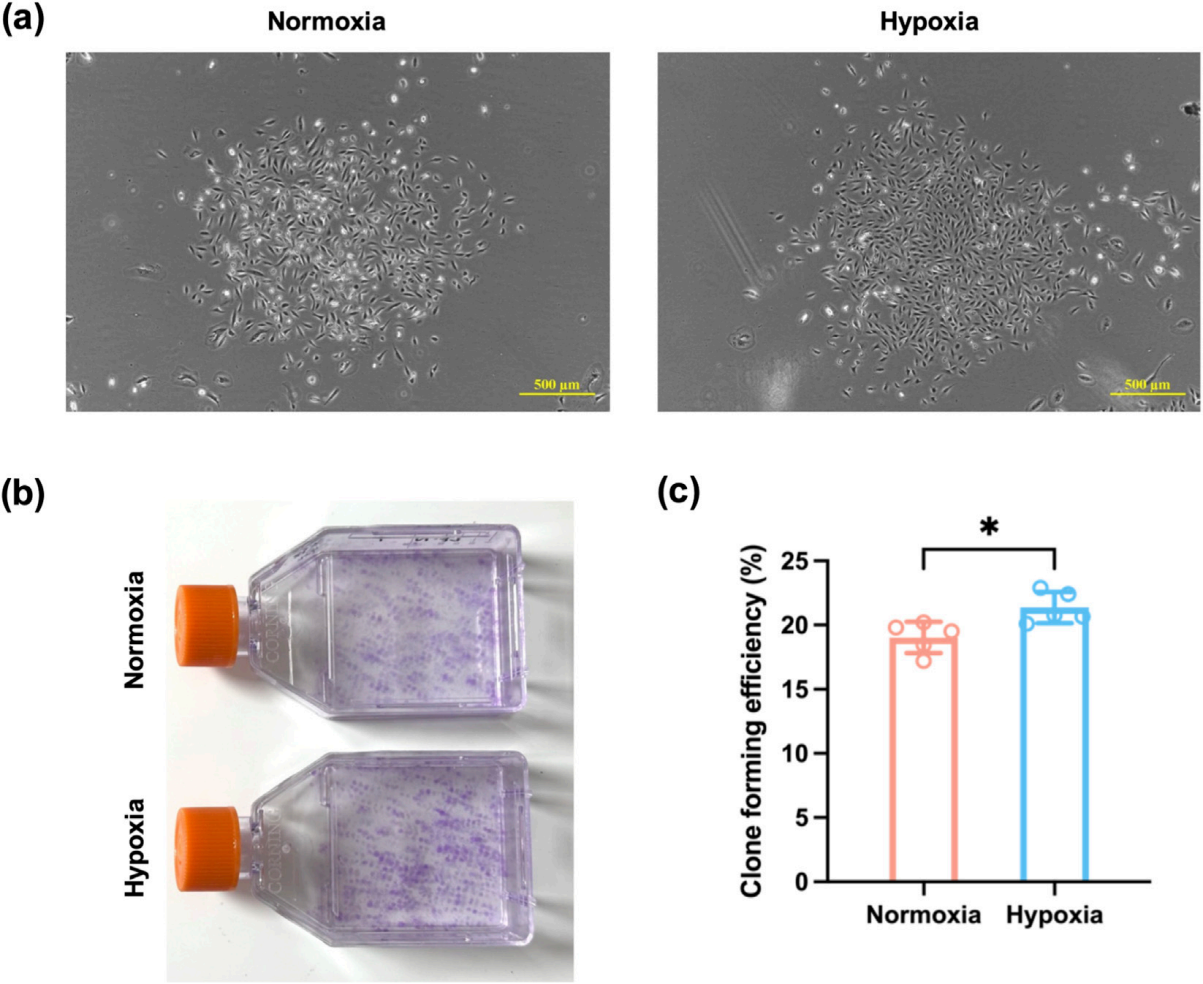
The effect of hypoxia on USC colony formation. **(a)** Bright field images of USC clones on day 7, scale bar: 500 μm; **(b)** Gross observation of flasks after crystal violet staining; **(c)** CFE of USCs, *p < 0.05.

Representative light microscopy images of colonies in the normoxia and hypoxia groups are shown in Figure 1a. Crystal violet staining further demonstrated a denser distribution of cell clones in the culture flasks of the hypoxic group, as shown in Figure 1b. After quantifying the number of cell clones, the colony formation efficiency (CFE) was calculated. The CFE in the hypoxia group was significantly higher than that in the normoxia group (21.36% ± 1.216% vs. 19.03% ± 1.211%, p < 0.05; Figure 1c). This difference in CFE suggests that hypoxia facilitates the formation of cell colonies in USCs.

### 3.2 Hypoxia does not affect cell viability and promotes the proliferation of USCs

**FIGURE 2:**
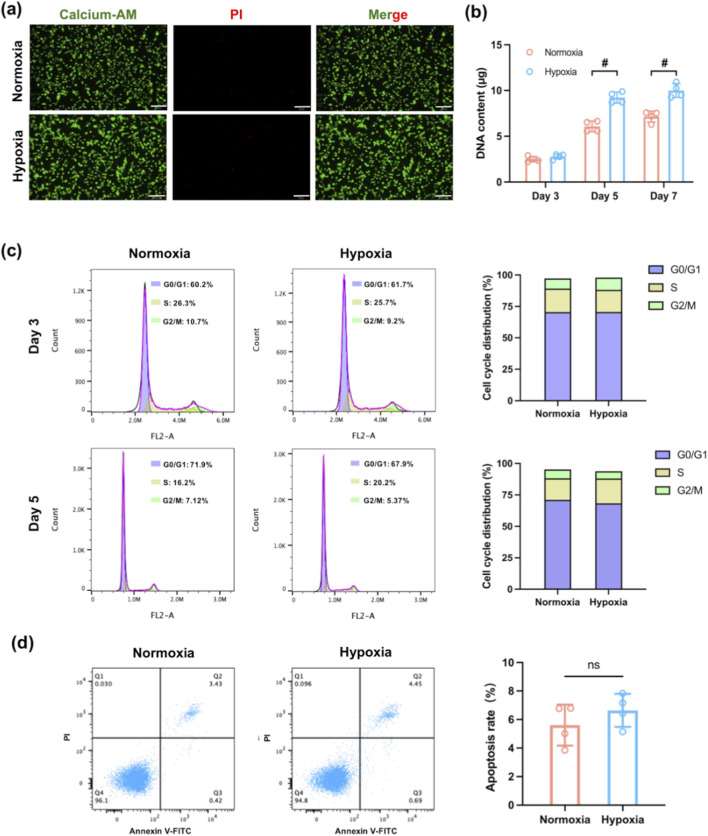
The effects of hypoxia on USC viability and proliferation. **(a)** Live/dead staining; green fluorescence: live cells; red fluorescence: dead cells; scale bar: 200 µm. **(b)** DNA content analysis, ^#^p < 0.01. **(c)** Cell cycle analysis; D3: day 3; D5: day 5. **(d)** Cell apoptosis analysis; ns: no significance.

To assess the effects of hypoxia on the viability and proliferation of USCs, a series of assays were conducted, including live/dead staining, cell cycle analysis, cell apoptosis assays, and DNA quantification. The live/dead staining results indicated a minimal presence of dead cells in both groups, suggesting that USCs maintained high viability under hypoxic conditions (Figure 2a). Cell cycle analysis showed comparable G0/G1-phase proportions between normoxic and hypoxic USCs on day 3. On day 5, hypoxic USCs exhibited a non-significant trend toward decreased G0/G1-phase cells (71.05% ± 2.06% vs. 68.30% ± 1.602%) and increased S/G2/M-phase cells (Figure 2c). Thus, the increased DNA content under hypoxia may reflect cumulative cell expansion rather than a significant shift in cell cycle distribution at the sampled time points. Furthermore, Annexin V-FITC/PI staining revealed a slight increase in total cell apoptosis in the hypoxia group (6.64% ± 1.15%) compared to the normoxia group (5.61% ± 1.44%), although this difference did not reach statistical significance (p > 0.05; Figure 2d).

The proliferation of USCs was evaluated through DNA content analysis. On day 3, the DNA content in the hypoxia group was marginally higher (2.78 ± 0.27 µg) compared to the normoxia group (2.49 ± 0.3 µg), although this difference was not statistically significant. However, from day 5 onward, a significant increase in DNA content was observed in the hypoxia group relative to the normoxia group (day 5: 9.22 ± 0.61 µg vs. 6.08 ± 0.58 µg, p < 0.01; day 7: 9.99 ± 0.75 µg vs. 7.14 ± 0.6 µg, p < 0.01; Figure 2b). These findings collectively suggest that hypoxia does not compromise the viability of USCs and significantly enhances their proliferation.

### 3.3 Hypoxia enhances the migration of USCs *in vitro*

**FIGURE 3:**
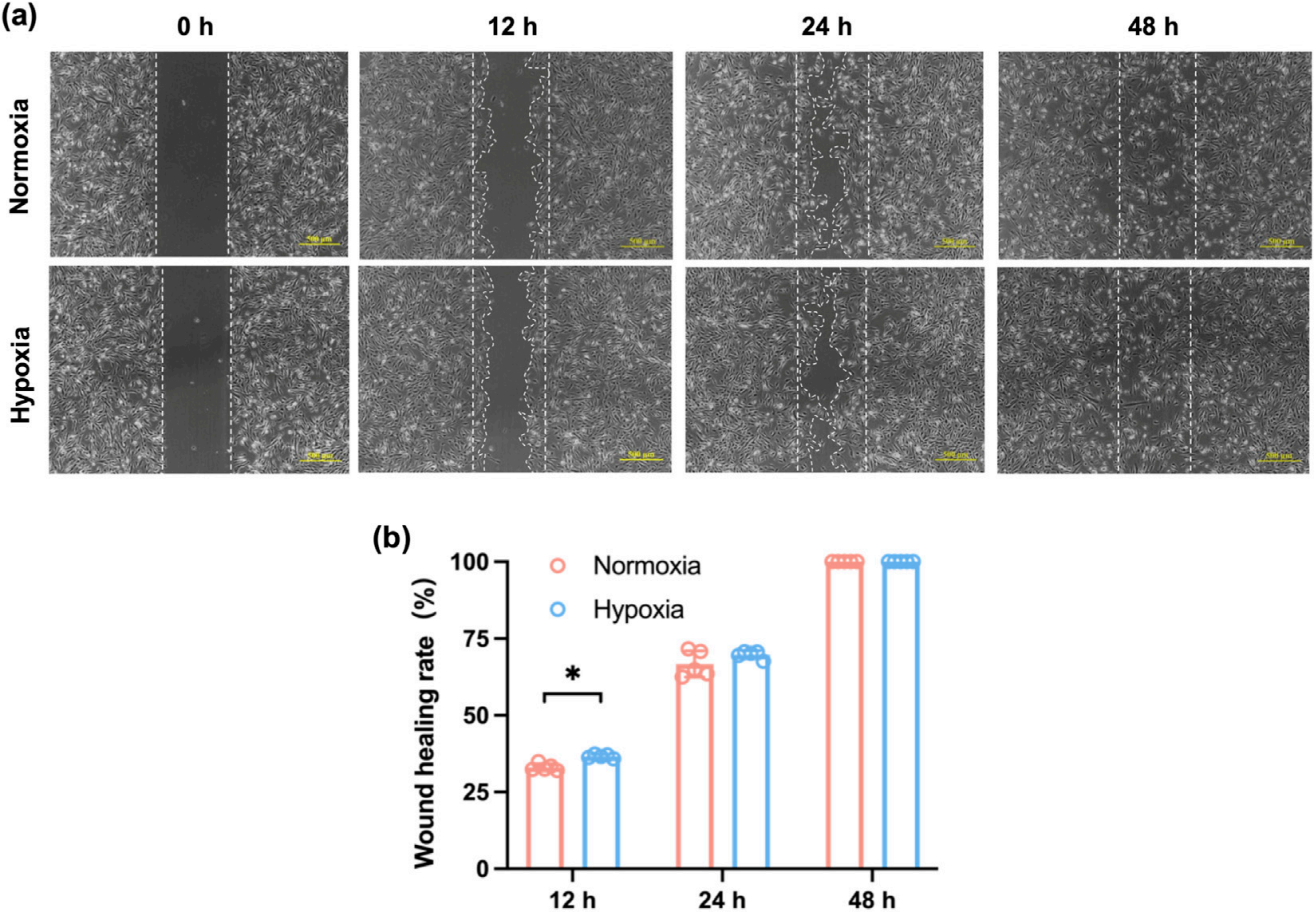
The effect of hypoxia on USC migration. **(a)** Microscopic images, scale bar: 500 μm; **(b)** Wound healing rates, *p < 0.05.

Cell migration was assessed using a scratch assay, with wound healing rates calculated at 0, 12, 24, and 48 h (Figure 3a). At 12 h, the hypoxia group demonstrated a significantly higher wound healing rate compared with the normoxia group (36.66% ± 0.63% vs. 33.08% ± 1.19%, p < 0.05). At 24 h, the hypoxia group continued to exhibit a higher wound-healing rate (69.77% ± 1.28% vs. 66.69% ± 4.28%, p > 0.05). By 48 h, both groups had achieved complete wound closure (Figure 3b). These findings suggest that hypoxia enhances the migration of USCs *in vitro*.

### 3.4 Hypoxia does not modify the phenotype of USCs but enhances stemness gene expression

**FIGURE 4:**
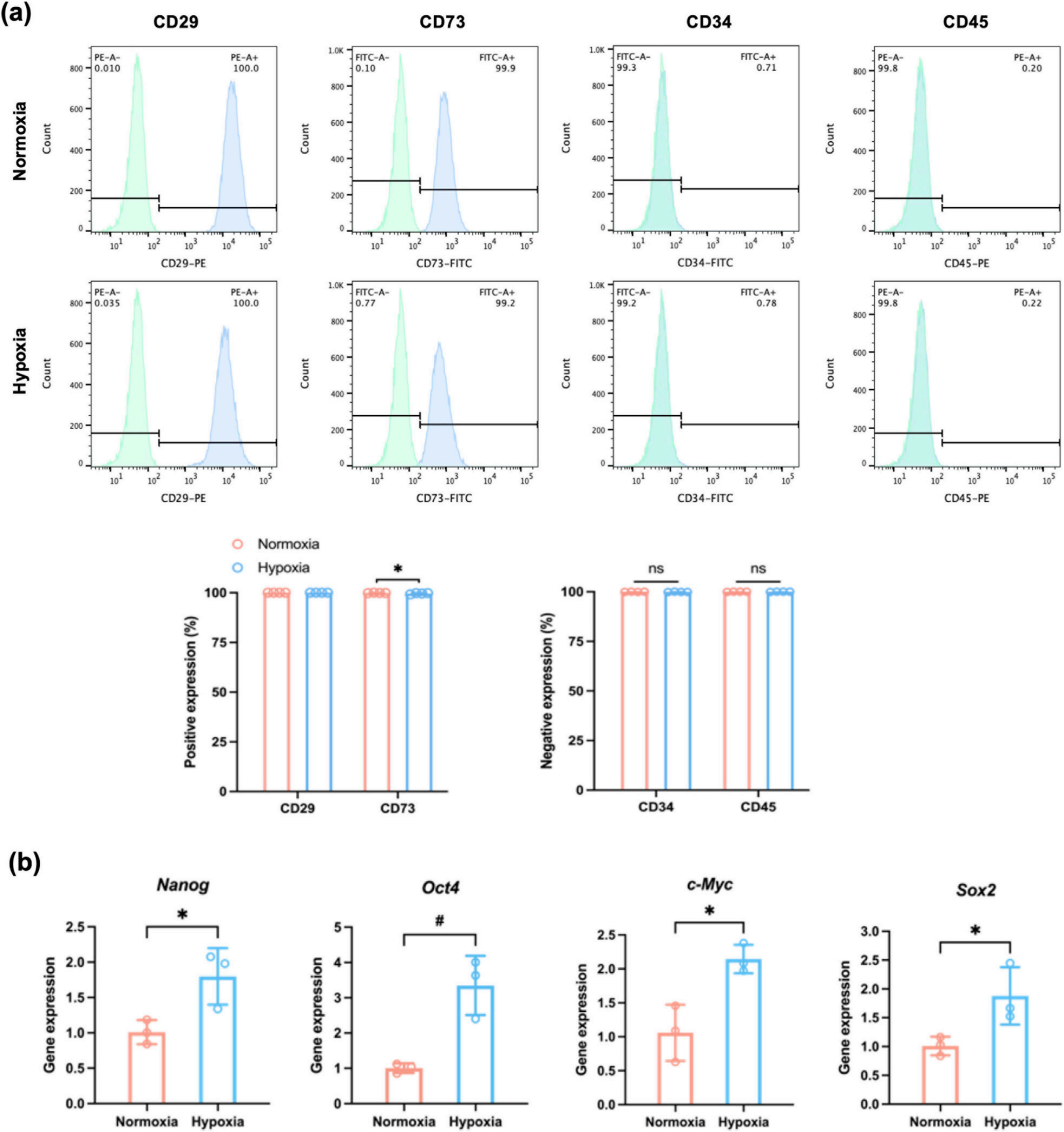
The impact of hypoxia on the phenotype and stemness-related gene expression of USCs. **(a)** Cell surface markers. **(b)** Stemness-related gene expression, *p < 0.05, ^#^p < 0.01.

To investigate whether hypoxia influences the phenotype of USCs, flow cytometry was employed to analyze the surface markers of USCs. Both the normoxic and hypoxic conditions exhibited comparable levels of positive markers (CD29 and CD73) and negative markers (CD34 and CD45) (Figure 4a). Additionally, the expression of stemness-related genes, including Nanog, Oct4, c-Myc, and Sox2, was evaluated. As illustrated in Figure 4b, the expression levels of all four stemness-related genes were significantly elevated in the hypoxia group compared to the normoxia group: Nanog (1.010 ± 0.1704 vs. 1.798 ± 0.4001, p < 0.05), Oct4 (1.006 ± 0.134 vs. 3.348 ± 0.841, p < 0.01), c-Myc (1.059 ± 0.414 vs. 2.146 ± 0.210, p < 0.05), and Sox2 (1.009 ± 0.162 vs. 1.879 ± 0.498, p < 0.05). These findings indicate that while hypoxia does not affect the surface marker expression of USCs, it significantly enhances the expression of stemness-related genes.

### 3.5 Hypoxia enhances the multi-lineage differentiation potential of USCs

**FIGURE 5:**
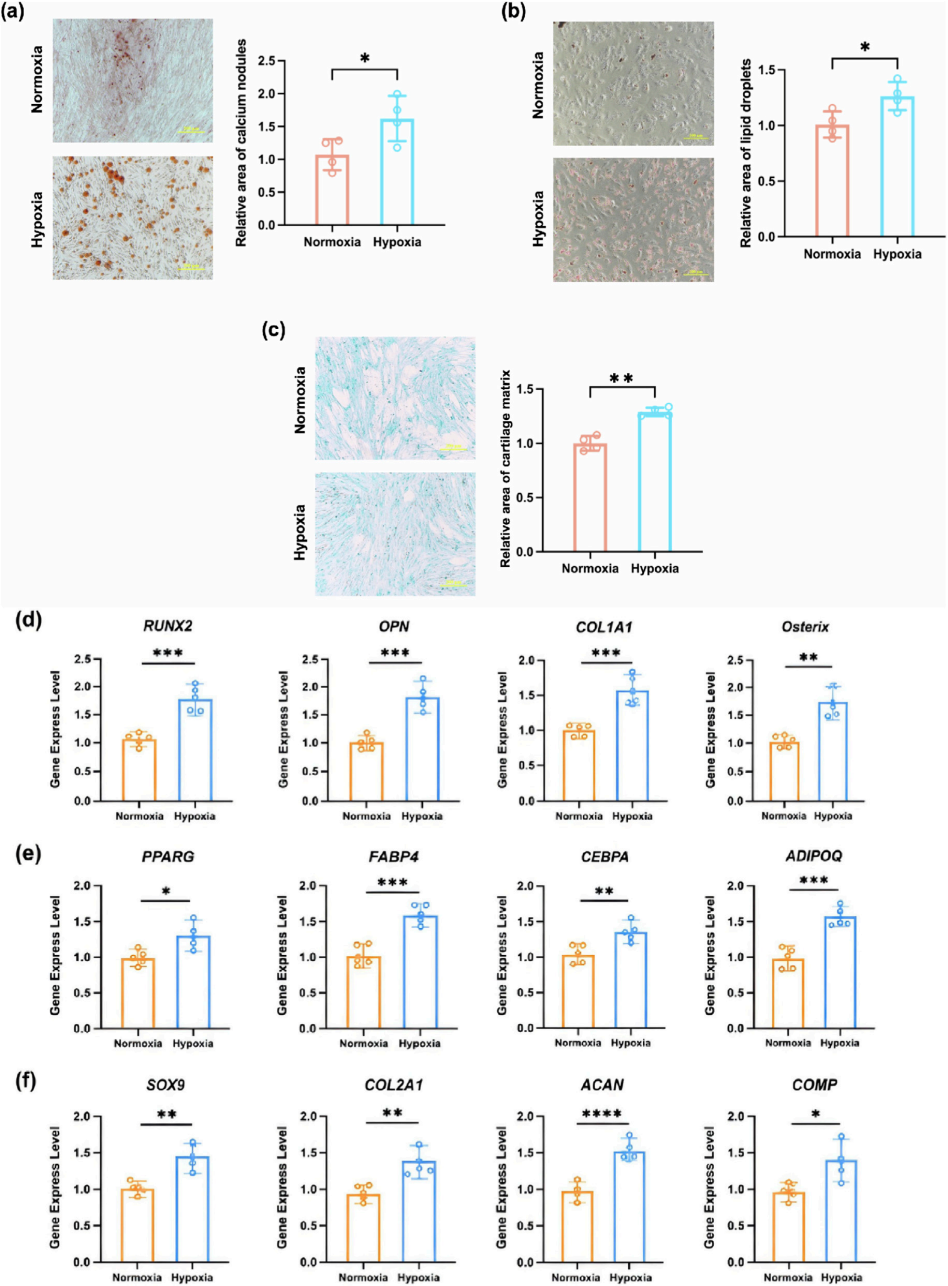
The effects of hypoxia on the osteogenic, adipogenic, and chondrogenic differentiation of USCs. **(a)** Alizarin Red staining for calcium nodules; scale bar: 200 μm; **(b)** Oil Red O staining for lipid droplets; scale bar: 100 μm; **(c)** Alcian Blue staining for cartilage matrix; scale bar: 200 µm. **(d)** qPCR analysis of osteogenic markers, including RUNX2, OPN, COL1A1, and Osterix. **(e)** qPCR analysis of adipogenic markers, including PPARG, FABP4, CEBPA, and ADIPOQ. **(f)** qPCR analysis of chondrogenic markers, including SOX9, COL2A1, ACAN, and COMP. Data are presented as mean ± SD, n = 5. *p < 0.05, **p < 0.01, ***p < 0.001, ****p < 0.0001.

To assess the impact of hypoxic conditions on the multi-lineage differentiation potential of USCs, cells were subjected to osteogenic, adipogenic, and chondrogenic differentiation under normoxic or hypoxic conditions. During osteogenic differentiation, the hypoxia group demonstrated a significantly larger area of Alizarin Red-positive calcium nodules compared to the normoxia group (1.621 ± 0.345 vs. 1.071 ± 0.237, p < 0.05; Figure 5a). Similarly, in adipogenic differentiation, Oil Red O staining revealed a significantly greater area of lipid droplet accumulation in the hypoxia group (1.264 ± 0.127 vs. 1.009 ± 0.118, p < 0.05; Figure 5b). Furthermore, during chondrogenic differentiation, Alcian Blue staining indicated a significantly increased area of cartilage matrix deposition in the hypoxia group (1.289 ± 0.039 vs. 1.000 ± 0.067, p < 0.01; Figure 5c).

To further validate the multilineage differentiation potential at the gene expression level, RT-qPCR was performed to examine lineage-specific markers after 14 days of induction periods. Compared with normoxic culture, hypoxia significantly upregulated the expression of osteogenic markers, including RUNX2, OPN, COL1A1, and Osterix (Figure 5d). In addition, the adipogenic markers PPARG, FABP4, CEBPA, and ADIPOQ were significantly increased in the hypoxia group compared with the normoxia group (Figure 5e). Similarly, the chondrogenic markers SOX9, COL2A1, ACAN, and COMP were significantly upregulated under hypoxic conditions (Figure 5f). Collectively, these staining and gene expression results demonstrate that hypoxia enhances the osteogenic, adipogenic, and chondrogenic differentiation of USCs.

### 3.6 Hypoxia influences the paracrine activity and transcriptome of USCs

**FIGURE 6:**
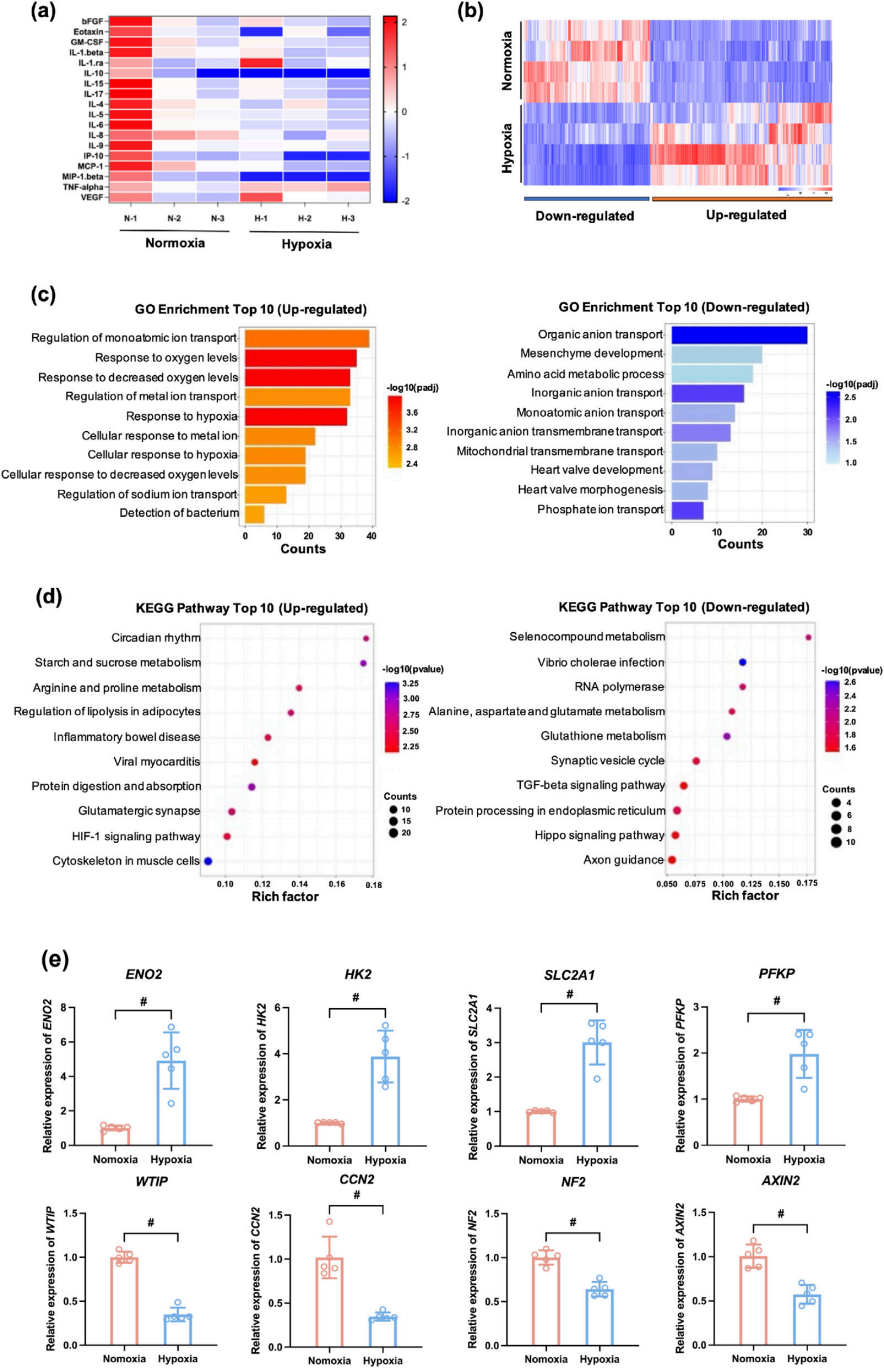
The impact of hypoxia on the paracrine and transcriptomic profile of USCs. **(a)** Heatmap depicting the secretion of cytokines; **(b)** Heatmap showing differential gene expression between hypoxic and normoxic conditions; **(c)** The top ten upregulated and downregulated GO terms in biological processes; **(d)** The top ten upregulated and downregulated KEGG pathways; **(e)** The expression of key genes in the HIF signaling pathway (*ENO2*, *HK2*, *SLC2A1*, and *PFKP*) and the Hippo signaling pathway (*WTIP*, *CCN2*, *NF2*, and *AXIN2*). n = 5 in each group. ^#^p < 0.01.

The cytokine secretion profiles of USCs cultured under normoxic or hypoxic conditions are detailed in Supplementary Table S1. Upon normalization of cytokine levels to DNA content, it was observed that the secretion of vascular endothelial growth factor (VEGF)—a critical growth factor in angiogenesis—was significantly elevated in the hypoxic group (Figure 6a). Notably, the secretion levels of other cytokines did not exhibit statistically significant differences between the hypoxic and normoxic groups (Supplementary Material; Supplementary Figure S1).

RNA sequencing identified 1,267 differentially expressed genes (748 upregulated and 519 downregulated) in USCs under hypoxia, indicating a comprehensive transcriptional response to hypoxic conditions (Figure 6b). GO enrichment analysis revealed that the upregulated genes were predominantly associated with ion transport and oxygen-sensing processes, particularly monoatomic ion transport, metal ion transport, response to oxygen levels, response to decreased oxygen levels, and hypoxia response. Conversely, the downregulated genes were primarily related to organic anion transport, mesenchyme development, and amino acid metabolism (Figure 6c).

The KEGG analysis revealed the activation of pathways associated with adaptive metabolism and circadian regulation, encompassing circadian rhythm, starch and sucrose metabolism, arginine and proline metabolism, lipolysis regulation in adipocytes, and HIF-1 signaling (Figure 6d). In contrast, hypoxic conditions led to the suppression of selenocompound metabolism, RNA polymerase activity, glutathione metabolism, TGF-β signaling, and the Hippo pathway (Figure 6d). Furthermore, RT-PCR was performed to validate the impact of hypoxia on the upregulated and downregulated KEGG pathways, respectively. The results demonstrated that, compared with the normoxia group, hypoxia significantly enhanced the expression of key genes in the HIF-1 signaling pathway (*ENO2*, *HK2*, *SLC2A1*, and *PFKP*), and inhibited the expression of genes associated with the Hippo signaling pathway (*WTIP*, *CCN2*, *NF2*, and *AXIN2*) (Figure 6e). These transcriptomic alterations suggest that hypoxia is associated with changes in oxygen sensing and metabolic adaptation in USCs, while the functional relevance of these changes requires further validation.

## 4 Discussion

Since their initial characterization in 2008, USCs have garnered significant attention due to their non-invasive isolation methods, robust proliferation capabilities, and potential for multilineage differentiation. Despite this growing interest, the biological characteristics of USCs under hypoxic conditions—an essential aspect of injured tissue microenvironments—remain inadequately understood. In this study, we demonstrate that hypoxia (3% O_2_) does not adversely affect the viability of USCs; rather, it significantly enhances their colony formation, proliferation, migration, and expression of genes associated with stemness. These findings align with previous research indicating that hypoxia promotes the proliferation and colony formation of USCs cultured on collagen type I scaffolds, primarily by increasing the proportion of cells in the S phase of the cell cycle (Chun et al., 2016). Similar effects have been observed in MSCs, where hypoxic conditions upregulate proliferation and the expression of stemness-related genes (Hu et al., 2023; Kheirandish et al., 2017; Obradovic et al., 2019; Kwon et al., 2017), without inducing apoptosis or significantly altering the cell cycle (Grayson et al., 2007; Rosová et al., 2008).

The influence of hypoxia on the differentiation of MSCs has been a subject of debate, with studies reporting a spectrum of outcomes, including inhibition (Boyette et al., 2014; Yang et al., 2011; Hsu et al., 2013), no obvious effect (Pezzi et al., 2017), and enhancement (Tsai et al., 2011; Kang et al., 2018; Leijten et al., 2014). These inconsistencies are likely attributable to variations in experimental culture conditions, particularly differences in oxygen tension. In the present study, both histochemical staining and RT-PCR analysis of lineage-specific markers showed that USCs cultured under 3% O_2_ exhibited enhanced osteogenic, chondrogenic, and adipogenic differentiation compared to those maintained under normoxia. This suggests that moderate hypoxia may favorably influence USC differentiation. Paracrine activity, a fundamental characteristic of stem cells, is vital for the efficacy of stem cell-based therapies. The cytokines released by stem cells have profound impacts on adjacent tissues, often assuming a more critical role than the differentiation of graft cells into the functional cells of injured tissues. Previous studies have reported heterogeneous effects of hypoxia on the paracrine secretion of stem/stromal cells, which may vary depending on oxygen tension, cell source, and culture conditions (Zhao et al., 2022; Zhang et al., 2020; Chang et al., 2013; Quade et al., 2020; Xiang and Xie, 2018; Chen et al., 2014). In the present study, although the secretion levels of most cytokines showed a decreasing trend without statistical significance (Supplementary Table S1), normalization to DNA content revealed a significant increase in VEGF secretion under 3% O_2_ (Figure 6a). In contrast, other factors, including IL-6, IL-8, and bFGF, were not significantly altered. These findings suggest that moderate hypoxic preconditioning does not globally enhance USC-derived trophic factor secretion, but instead induces a more selective, VEGF-dominant pro-angiogenic secretory response. This pattern differs from some reports in bone marrow-derived MSCs, in which multiple growth factors are upregulated under hypoxia. Further studies comparing different oxygen tensions will be necessary to determine whether distinct hypoxic conditions elicit broader or more selective paracrine responses in USCs.

To further investigate the cellular response to hypoxia, transcriptomic profiling was conducted, identifying 1,267 differentially expressed genes. GO analysis indicated an enrichment of biological processes associated with oxygen sensing, including responses to hypoxia and ion transport. In contrast, downregulated genes were associated with organic anion transport, mesenchyme development, and amino acid metabolism, suggesting a potential metabolic shift under hypoxic conditions. KEGG analysis revealed the upregulation of adaptive and metabolic pathways, such as circadian rhythm regulation, amino acid metabolism, and notably, the HIF-1 signaling pathway (Figures 6d,e). This finding is consistent with previous studies that have demonstrated HIF-1 activation as a key mechanism underlying enhanced cell survival and regenerative capacity in MSCs under hypoxic conditions (Mahjoor et al., 2023; Ejtehadifar et al., 2015; Okuyama et al., 2006; Beegle et al., 2015). The observed downregulation of the Hippo signaling pathway (Figures 6d,e), a known negative regulator of cell proliferation and tissue regeneration (Wu et al., 2003), further supports our observation that hypoxia is associated with increased USC expansion and may contribute to functional changes relevant to tissue repair.

It should also be noted that, although hypoxia significantly increased the scratch healing rate at 12 h, the magnitude of this difference was modest. Therefore, this result should be interpreted as a modest improvement in early USC migration *in vitro*, and its biological relevance requires further validation using additional functional assays and *in vivo* models.

Furthermore, tumorigenicity represents a critical safety concern in the clinical application of stem cells. Accumulating evidence from MSC studies indicates that hypoxic culture conditions enhance stemness-associated features without conferring tumorigenic potential. In particular, adipose-derived MSCs cultured under hypoxia have been shown to exhibit elevated expression of pluripotency-related factors while failing to induce tumor formation in both *in vitro* and *in vivo* models (Feng et al., 2014). Consistent with these observations, previous studies by Chun et al. demonstrated that USCs implanted into mouse kidneys did not form teratomas, suggesting that despite their stem cell-like properties, these cells lack intrinsic tumorigenic capacity (Chun et al., 2012). In the subsequent work, they reported that prolonged expansion of USCs under hypoxic conditions preserved genomic stability, maintained a normal karyotype, and did not lead to tumor formation (Kwon et al., 2017). In this study, our results showed that hypoxia upregulated the expression of stemness genes, including *Nanog*, *Oct4*, and *Sox2*. Importantly, in our previous study, hypoxia-preconditioned USC-seeded tissue engineered constructs developed to enhance wound healing did not exhibit tumorigenic behavior following implantation *in vivo* (Zhang et al., 2020). Together, these data support the interpretation that hypoxia-induced activation of stemness-related programs reflects a functional adaptation toward stemness maintenance, rather than malignant transformation, and does not indicate an increased tumorigenic risk.

Despite the insights gained, this study has limitations. Firstly, this study assessed the impact of hypoxia on USCs at an oxygen concentration of 3%, selected as a moderate and physiologically relevant hypoxic condition based on prior research. However, alternative oxygen concentrations, such as 1% or 5%, were not investigated, leaving it unclear whether the effects observed are specific to the 3% condition or indicative of a more general hypoxia-dependent response. Additionally, while oxygen tension was regulated at the gas phase level, the precise oxygen concentration within the culture medium was not directly measured. This limitation is common in hypoxia-related *in vitro* studies and may introduce variability in interpreting oxygen-dependent effects. Future research incorporating a range of oxygen tensions and direct measurement of medium oxygen levels will be crucial in elucidating the dose-dependent effects of hypoxia on USC biology. Secondly, the study was conducted exclusively *in vitro*, without investigating the potential applications of hypoxia-preconditioned USCs *in vivo*. Thirdly, USCs were exclusively isolated from healthy male donors. This selection criterion was implemented to mitigate the risk of sample contamination during urine collection, which is more prevalent in female donors due to anatomical considerations. The findings demonstrated general consistency across donors; however, the relatively small sample size may not adequately represent donor-to-donor variability. Furthermore, the restriction of donors to a relatively young demographic limits the generalizability of the results. Previous studies have indicated that donor sex and age can influence the biological properties of stem cells (Maged et al., 2024; Selle et al., 2022). Consequently, the findings of this study may not be directly applicable to female donors or older populations. Future research involving donors of both sexes and a wider age range is necessary to more comprehensively assess the variability of USCs under hypoxic conditions. In addition, the current findings may not fully capture donor-to-donor variability, which warrants further investigation in larger and more heterogeneous donor populations. Moreover, the upregulation of the HIF-1 signaling pathway and the downregulation of the Hippo pathway observed in the current transcriptomic and RT-PCR analyses should be interpreted as preliminary and descriptive findings. Whether these molecular alterations can directly promote angiogenesis or enhance wound-healing capacity remains to be determined and requires further experimental validation. Loss-of-function and rescue experiments are needed to determine whether these pathways are required for the hypoxia-induced functional improvement of USCs, and this will be further investigated in future studies. Considering the efficacy of USCs in repairing the urinary tract (Wu et al., 2011; Tu et al., 2022), musculoskeletal system (Liu et al., 2021; Guan et al., 2015) and cardiovascular system (Dong et al., 2016), future *in vivo* studies may further explore the therapeutic potential of hypoxia-preconditioned USCs in these disease-relevant models. Such investigations would help to elucidate the effects of hypoxia on the *in vivo* biological behavior of USCs in an application-specific manner, incorporating protein-level validation, and integrating functional assays—such as angiogenesis and wound-healing models—to better link hypoxia-induced molecular alterations with regenerative outcomes and further determine the effects of hypoxia on the regenerative capacity of USCs, thereby facilitating their clinical translation.

## 5 Conclusion

In this study, the incubation of USCs under hypoxia (3% O_2_) was found to maintain cell viability while enhancing colony formation, proliferation, and migration capabilities. Furthermore, hypoxia upregulated the expression of stemness-related genes and facilitated the differentiation of USCs into osteogenic, adipogenic, and chondrogenic lineages. The study also demonstrated that hypoxia increased VEGF secretion by USCs. Transcriptome sequencing analysis indicated that hypoxia induced reprogramming in USCs, characterized by the upregulation of oxygen-sensing, metabolic, and HIF-1 pathways, alongside the downregulation of the Hippo signaling pathway. These findings elucidate the complex effects of hypoxia on USC biology and provide a foundation for further investigation into the potential applications of USCs in regenerative medicine.

## Data Availability

The raw data supporting the conclusions of this article will be made available by the authors, without undue reservation.
