# Supplementary material for: Effects of hypoxia on the biological characteristics of urine-derived stem cells
Source: Front Cell Dev Biol. 2026 Jul 27;14:1851623. doi: 10.3389/fcell.2026.1851623 (PMC13454028; doi:10.3389/fcell.2026.1851623)
Supplement: Supplementary file 1 [file DataSheet1.docx]

Supplementary Material

**Table S1. DNA and Growth Factor Content**

| **Sample** | **DNA** | **bFGF** | **Eotaxin** | **G-CSF** | **GM-CSF** | **IFN-γ** | **IL-1.beta** | **IL-1.ra** | **IL-10** | **IL-12** | **IL-13** | **IL-15** | **IL-17** | **IL-2** |
| --- | --- | --- | --- | --- | --- | --- | --- | --- | --- | --- | --- | --- | --- | --- |
| N-1 | 4.955 | 169 | 12 | 119 | 389.5 | OOR < | 24 | 691.5 | 12.5 | OOR < | OOR < | OOR < | 77.5 | OOR < |
| N-2 | 10.255 | 142 | 12 | OOR < | 391.5 | OOR < | 24 | 395.5 | 12.5 | OOR < | OOR < | OOR < | 46.3 | OOR < |
| N-3 | 11.615 | 82 | 12 | OOR < | 275.5 | OOR < | 24 | 691.5 | OOR < | OOR < | OOR < | OOR < | 25.5 | OOR < |
| H-1 | 5.33 | 82 | OOR < | OOR < | 179 | OOR < | 12.5 | 1164.5 | OOR < | OOR < | OOR < | OOR < | 25.5 | OOR < |
| H-2 | 9.23 | 82 | 12 | OOR < | 235.5 | OOR < | 12.5 | 395.5 | OOR < | OOR < | OOR < | OOR < | 25.5 | OOR < |
| H-3 | 8.125 | 47 | 3 | OOR < | 174 | OOR < | 12.5 | 691.5 | OOR < | OOR < | OOR < | OOR < | 4 | OOR < |

bFGF, basic fibroblast growth factor; Eotaxin, eosinophil chemotactic protein; G-CSF, granulocyte colony-stimulating factor; GM-CSF, granulocyte-macrophage colony-stimulating factor; IFN-γ, interferon gamma; IL-1.beta, interleukin-1 beta; IL-1.ra, interleukin-1 receptor antagonist; IL-10, interleukin-10; IL-12, interleukin-12; IL-13, interleukin-13; IL-15, interleukin-15; IL-17, interleukin-17; IL-2, interleukin-2; N: Normoxia; H: Hypoxia; OOR<: If the concentration of the indicator is too low, falling outside the detection range of the standard curve, the result may be unreliable. Units: DNA: μg; Growth Factors: pg.

**Table S1. DNA and Growth Factor Content (continued)**

| **Sample** | **IL-4** | **IL-5** | **IL-6** | **IL-7** | **IL-8** | **IL-9** | **IP-10** | **MCP-1** | **MIP-1.alpha** | **MIP-1.beta** | **PDGF-BB** | **RANTES** | **TNF-alpha** | **VEGF** |
| --- | --- | --- | --- | --- | --- | --- | --- | --- | --- | --- | --- | --- | --- | --- |
| N-1 | 66.5 | 4442.5 | 49609.5 | OOR < | 8417.5 | 3969 | 365 | 1664.5 | 8.5 | 733 | OOR < | OOR < | 325.5 | 12019.5 |
| N-2 | 71 | 4033 | 38403.5 | OOR < | 14859 | 3435.5 | 284.5 | 1989.5 | OOR < | 661.5 | OOR < | OOR < | 511 | 11238.5 |
| N-3 | 71 | 3950.5 | 42968.5 | OOR < | 14612 | 3685.5 | 284.5 | 1502.5 | OOR < | 677 | 932 | OOR < | 511 | 11080.5 |
| H-1 | 21.5 | 1656 | 14532 | OOR < | 5038.5 | 1763 | 172 | 712.5 | OOR < | 367.5 | OOR < | OOR < | 325.5 | 15021.5 |
| H-2 | 62.5 | 3034.5 | 26252.5 | OOR < | 5213.5 | 3110.5 | OOR < | 649 | OOR < | 540.5 | OOR < | OOR < | 126 | 13242 |
| H-3 | 33 | 2007.5 | 19308.5 | OOR < | 8475 | 2299 | OOR < | 583 | OOR < | 545.5 | OOR < | OOR < | 325.5 | 11080.5 |

IL-4, interleukin-4; IL-5, interleukin-5; IL-6, interleukin-6; IL-7, interleukin-7; IL-8, interleukin-8; IL-9, interleukin-9; IP-10, interferon gamma-induced protein 10; MCP-1, monocyte chemoattractant protein-1; MIP-1.alpha, macrophage inflammatory protein-1 alpha; MIP-1.beta, macrophage inflammatory protein-1 beta; PDGF-BB, platelet-derived growth factor-BB; RANTES, regulated on activation, normal T cell expressed and secreted; TNF-alpha, tumor necrosis factor alpha; VEGF, vascular endothelial growth factor; N: Normoxia; H: Hypoxia; OOR<: If the concentration of the indicator is too low, falling outside the detection range of the standard curve, the result may be unreliable. Units: DNA: μg; Growth Factors: pg.

**Fig. S1.** The secretion of cytokines after normalization to DNA content.
